# Impact of Nirsevimab on RSV and Non‐RSV Severe Respiratory Infections in Hospitalized Infants

**DOI:** 10.1111/irv.70105

**Published:** 2025-04-29

**Authors:** María Luz García‐García, Patricia Alonso‐López, Sonia Alcolea, M. Arroyas, Francisco Pozo, Inmaculada Casas, Maria Iglesias‐Caballero, Rocío Sánchez‐León, Jara Hurtado‐Gallego, Cristina Calvo

**Affiliations:** ^1^ Pediatrics Department Severo Ochoa University Hospital Leganés Spain; ^2^ Puerta de Hierro Institute for Health Research (IDIPHISA) Majadahonda Spain; ^3^ Center for Biomedical Research in the Infectious Diseases Network (CIBERINFEC) Instituto de Salud Carlos III (ISCIII) Madrid Spain; ^4^ Reference and Research Laboratory for Respiratory Virus National Centre for Microbiology, Instituto de Salud Carlos III (ISCIII) Majadahonda Madrid Spain; ^5^ CIBER de Epidemiología y Salud Pública (CIBERESP) Instituto de Salud Carlos III (ISCIII) Madrid Spain; ^6^ Pediatric Infectious and Tropical Diseases Department La Paz University Hospital. La Paz Institute for Health Research (IdiPAZ), P° de la Castellana Madrid Spain; ^7^ European Society For Paediatric Infectious Diseases (ESPID) Seafield UK

**Keywords:** bronchiolitis, nirsevimab, respiratory infections, respiratory syncytial virus

## Abstract

**Background:**

Nirsevimab, a monoclonal antibody providing passive immunity against RSV infections in infants, was introduced in Spain in October 2023 for children under 6 months and those born during the epidemic season. This study aimed to compare the clinical and virological characteristics of respiratory infections in hospitalized infants before and after nirsevimab introduction.

**Methods:**

We carried out a prospective study across two hospitals in Madrid during the 2022–2023 and 2023–2024 epidemic seasons. The study included infants under 12 months of age that were hospitalized with lower respiratory tract infections (LRTIs). Clinical, epidemiological, and virological data were analyzed and compared between the periods before and after the introduction of nirsevimab, as well as according to whether the infants had received this preventive treatment.

**Results:**

A total of 669 infants were included: 480 from October 2022 to March 2023 (S1) and 189 from October 2023 to March 2024 (S2). Respiratory infection–related admissions decreased by 62.5% in S2, with a 74.5% reduction in ICU admissions. RSV‐related admissions decreased by 78%, HMPV by 36.6%, and adenovirus by 69.5%. Infants in S2 were older (*p* = 0.001) and had shorter hospital stays (*p* < 0.001) than in S1. Of 63 (33%) infants in S2 who received nirsevimab, 11 (17%) were diagnosed with RSV. High‐flow oxygen use was less frequent among RSV patients treated with nirsevimab (*p* = 0.002).

**Conclusions:**

Nirsevimab introduction was significantly associated with reduced hospitalizations and severity of RSV and other respiratory infections. Its use was associated with fewer admissions and reduced need for intensive care, especially in RSV‐infected infants but also in HMPV and adenovirus‐infected infants.

## 1 Introduction

Respiratory syncytial virus (RSV) is one of the most common seasonal respiratory infections, causing significant morbidity and mortality worldwide, particularly in children under the age of two [1, 2]. In Europe, the incidence of RSV‐associated hospitalization in the first year of life has been reported at 1.8% [3]. Nearly half of all hospitalizations due to acute lower respiratory tract infections (LRTIs) in the first year of life were associated with RSV, with the highest burden observed in infants under 3 months old, where the incidence rate reaches 3.3 per 1000 infant‐months.

In 1998, the Food and Drug Administration approved palivizumab (Synagis) for the prevention of severe LRTI secondary to RSV in pediatric patients at high risk for developing severe disease. In the United States, the use of palivizumab for prophylaxis is further limited by the recommendations of the American Academy of Pediatrics: infants born at less than 29 weeks of gestation entering their first RSV season, as well as select infants with congenital lung disease of prematurity or hemodynamically significant congenital heart disease during their first and second years of life [4]. However, more than 90% of patients admitted for RSV‐LRTI are otherwise healthy children who would not be eligible to receive palivizumab prophylaxis [1].

In July 2023, the Food and Drug Administration approved the monoclonal antibody nirsevimab (Beyfortus) for RSV prophylaxis. Shortly after, the Advisory Committee on Immunization Practices and the American Academy of Pediatrics unanimously recommended that all infants under 8 months of age entering their first RSV season receive nirsevimab, as well as infants at risk for severe RSV during their second year of life [5]. In Spain, nirsevimab has been administered in maternity wards from October 2023 to March 2024, as well as in an outpatient program to infants born between April 1, 2023, and October 1, 2023. In the Autonomous Community of Madrid, at the end of the campaign, the overall immunization coverage was 87% and 95% for those born during the transmission season [6].

The main objective of this study was to compare the clinical, virological, and epidemiological characteristics of severe respiratory infections associated with RSV and other respiratory viruses in children under 12 months, before and after the introduction of nirsevimab.

## 2 Material and Methods

This is a substudy of an ongoing prospective investigation into respiratory tract infections in children, funded by the Spanish Health Research Fund (FIS) and approved by the Medical Ethics Committee, at Severo Ochoa University Hospital and La Paz University Hospital. The study design was prospective, observational, and comparative cross‐sectional.

### 2.1 Study Population

The study population included all children under 12 months of age hospitalized for a LRTI in Severo Ochoa and La Paz Hospital (Madrid, Spain), during two epidemic periods: from October 1, 2022, to March 31, 2023 (S1), and from October 1, 2023, to March 31, 2024 (S2). Informed consent was obtained from parents or legal guardians.

### 2.2 Study Procedures

Clinical and epidemiological characteristics of patients were prospectively collected at admission. Data of all patients were compared based on whether they were admitted before or after the introduction of nirsevimab and whether they actually received it or not, according to the data recorded in SISPAL, the Public Health Information System of the Community of Madrid.

Acute expiratory wheezing was considered to be bronchiolitis when it occurred for the first time in children aged less than 2 years following the McConnochie [7] classical criteria. All other episodes of acute expiratory wheezing were considered to be recurrent wheezing. Asthma was diagnosed when it was the third documented episode of wheezing by a physician [8]. Laryngitis was related to inspiratory dyspnea without wheezing. Cases with both focal infiltrates and consolidation in chest x‐rays were, in the absence of wheezing, classified as pneumonia.

### 2.3 Virological Study

Specimens consisted of nasopharyngeal aspirates taken from each patient at admission and sent for virological investigation to the Influenza and Respiratory Viruses Laboratory at the National Center for Microbiology (ISCIII), Madrid, Spain. Three RT‐nested PCR assays to detect RSV, rhinovirus (HRV), human metapneumovirus (HMPV), human bocavirus (HBoV), adenovirus (AdV), influenza virus, parainfluenza virus (PIV), and human coronaviruses 229E, OC43, NL63, and HKU1 were performed [6]. An additional RT‐PCR assay was used to detect SARS‐CoV‐2. All the human coronaviruses have been grouped in this study and identified as HCOV. Viral coinfection was defined as the simultaneous detection of more than one respiratory virus in a single patient sample, regardless of the specific viruses identified.

### 2.4 Statistical Analysis

Descriptive data were presented as mean ± standard deviation or median with interquartile range for continuous variables and as counts and percentages for categorical variables. Continuous variables were compared using *T*‐tests or Mann–Whitney *U* tests, while categorical variables were compared using the chi‐squared test or Fisher's exact test, as applicable. Statistical significance was set at a *p* value of less than 0.05. All analyses were two‐tailed and conducted using IBM SPSS Statistics, Version 25 (IBM Corp., Armonk, New York, United States).

## 3 Results

**FIGURE 1:**
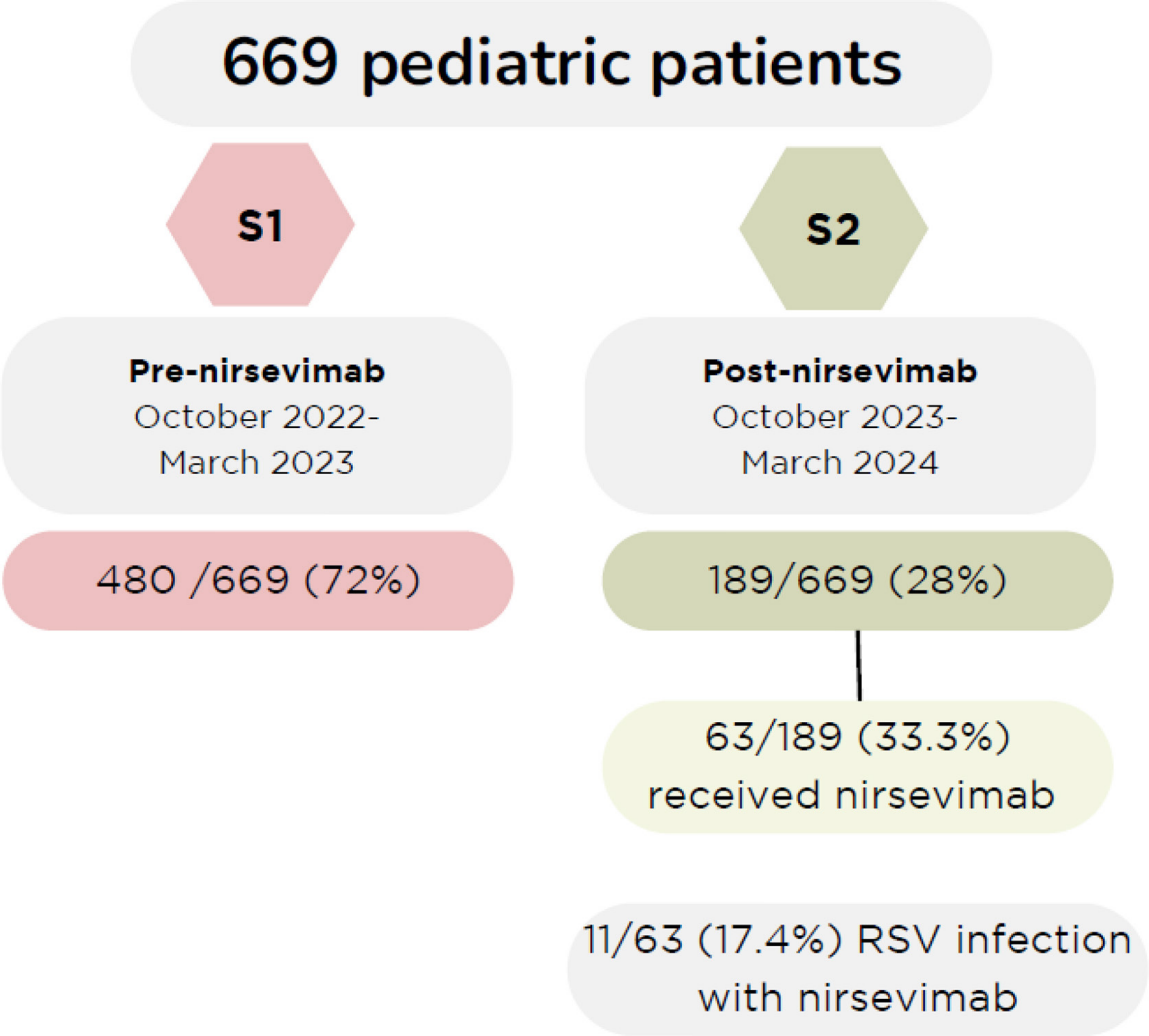
Number of infants less than 12 months of age admitted for lower respiratory tract infection before (S1) and after the introduction of nirsevimab in Spain (S2).

A total of 669 patients were included in the study. The number of admissions for LRTIs from October 2023 to March 2024 decreased by 62.5% compared to the same period in the previous year, with 480 infants admitted during S1 and 189 during S2 (Figure 1). RSV admissions decreased by 78% (336 in S1 vs. 73 in S2). In Season 2, 63 infants (33.3%) received nirsevimab, among whom 11 (17.4%) tested positive for RSV at admission. The mean age at nirsevimab administration was 36.6 ± 57.9 days, and the mean interval between administration and hospital admission was 60.8 ± 41 days.

### 3.1 Comparison of Clinical and Virological Characteristics of Respiratory Infections Before (S1, *N* = 480) and After (S2, *N* = 189) the Introduction of Nirsevimab

#### 3.1.1 Clinical Features

Table 1 summarizes the main clinical and virological characteristics of respiratory infections during the two periods, pre‐ and postnirsevimab. The diagnosis of bronchiolitis was less frequent in S2 compared to S1 (67% vs. 78%, *p* = 0.002). Patients in S2 were significantly older (*p* = 0.001), with a lower proportion of infants younger than 1 month of age (*p* = 0.001).

**TABLE 1:** Comparison of clinical and virological characteristics of respiratory infections in infants before and after the introduction of nirsevimab prophylaxis (S1/prenirsevimab: October 1, 2022, to March 31, 2023) (S2/postnirsevimab: October 1, 2023, to March 31, 2024).

	Postnirsevimab (S2)	Prenirsevimab (S1)	*p* value	Odds ratio (95% confidence interval)
	*N* = 189	*N* = 480		
Clinical features				
Sex (male)	1215 (64%)	308 (64%)	0.972	0.9 (0.7–1.4)
Prematurity	28 (18%)	64 (16%)	0.594	1.1 (0.7–1.9)
Age (months)*	5.1 ± 3.5	4.1 ± 3.3	0.001	
Age < 1 month	14 (7%)	85 (18%)	0.001	0.4 (0.2–0.7)
Fever ≥ 38°C	88 (46.6%)	212 (44%)	0.575	1.1 (0.8–1.5)
Duration of fever (days)*	2.7 ± 2.1	2.8 ± 2.1	0.701	
Hypoxia, oxygen saturation < 95%	157 (84%)	408 (86%)	0.566	0.9 (0.6–1.4)
Duration of hypoxia (days)*	4.2 ± 2.5	5.3 ± 3.9	0.173	
Duration of hospital stay (days)*	4.5 ± 2.7	5.8 ± 4.0	< 0.001	
Duration of stay > 5 days	54 (29%)	211 (44%)	< 0.001	0.5 (0.3–0.7)
Duration of stay > 5 days (younger 3 months)	23 (29%)	130 (54%)	< 0.001	0.3 (0.2–0.6)
Duration of stay > 5 days (younger 6 months)	41 (33%)	174 (49%)	0.003	0.5 (0.3–0.8)
High oxygen flow	105 (56%)	292 (61%)	0.208	0.8 (0.6–1.1)
Duration of high oxygen flow (days)*	4.2 ± 2.2	4.7 ± 3.1	0.130	
Intensive care unit admission	30 (16%)	118 (25%)	0.017	0.6 (0.4–0.9)
Duration of intensive care unit admission (days)*	3.5 ± 1.5	3.9 ± 2.8	0.373	
Mechanical ventilation	26 (14.9%)	98 (21%)	0.090	0.7 (0.4–1.1)
Duration of mechanical ventilation (days)*	2.4 ± 1.9	3.3 ± 2.5	0.114	
Antimicrobial treatment	56 (30%)	148 (32%)	0.688	0.9 (0.6–1.3)
Chest infiltrate	51 (27%)	140 (30%)	0.460	0.9 (0.6–1.0)
Diagnosis				
Bronchiolitis	116 (67%)	352 (78%)	0.002	
Recurrent wheezing	39 (23%)	58 (13%)		
Pneumonia	16 (9%)	42 (9%)		
Laryngitis	2 (1%)	2 (0.3%)		
Viral detection				
RSV	73 (39%)	336 (71%)	< 0.001	0.3 (0.2–0.4)
HRV	56 (30%)	53 (11%)	< 0.001	3.4 (2.2–5.1)
HBoV	9 (5%)	7 (1.5%)	0.013	3.3 (1.2–9.1)
HMPV	19 (10%)	30 (6%)	0.080	1.7 (0.9–3.1)
AdV	7 (4%)	23 (5%)	0.526	0.7 (0.3–1.8)
Influenza	7 (4%)	14 (3%)	0.614	1.2 (0.5–3.2)
PIV	10 (5%)	14 (3%)	0.132	1.9 (0.8–4.3)
HCoV	11 (6%)	24 (5%)	0.687	1.2 (0.6–2.4)
Coinfection	24 (14%)	61 (11%)	0.261	1.2 (0.8–1.9)
Enterovirus	0	12 (2%)		
Overall viral detection	147 (83%)	444 (94%)	< 0.001	0.3 (0.2–0.5)

Abbreviations: AdV, adenovirus; EV, enterovirus; HBoV, human bocavirus; HCoV, human coronavirus; HMPV, human metapneumovirus; HRV, rhinovirus; PIV, parainfluenza virus; RSV, respiratory syncytial virus.

* Mean and standard deviation.

The mean length of hospital stay was significantly reduced during S2 (*p* < 0.001), with a notable decrease in the proportion of hospitalizations exceeding 5 days (*p* < 0.001). Specifically, the likelihood of hospital stays longer than 5 days decreased by 64.6% in infants younger than 3 months (*p* < 0.001) and by 47.7% in those younger than 6 months (*p* = 0.0034) during S2 compared to S1. Similarly, admissions to the intensive care unit (ICU) declined significantly, from 118 cases in S1 to 30 cases in S2 (74.5% reduction, *p* = 0.017).

**FIGURE 2:**
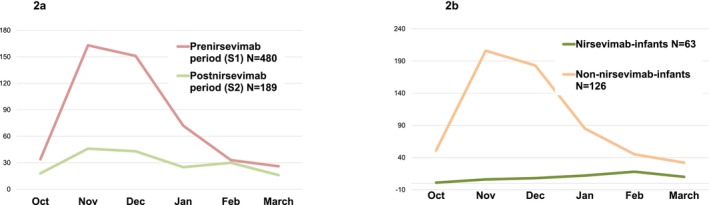
(a) Monthly distribution of respiratory admissions between October 1, 2022, and March 31, 2023 (S1/prenirsevimab) and October 1, 2023, and March 31, 2024 (S2/postnirsevimab). (b) Monthly distribution of respiratory admissions between October 1, 2022, and March 31, 2023 (S1/prenirsevimab), and October 1, 2023, and March 31, 2024 (S2/postnirsevimab), in infants who received nirsevimab and those who did not.

Monthly admission patterns remained comparable between S1 and S2, with peak cases primarily observed in November and December (Figure 2a).

#### 3.1.2 Viral Detection

In S2, the overall viral detection rate was significantly lower compared to S1 (147 cases vs. 444, 67% reduction), as was the detection of RSV (73 cases vs. 336, 78% reduction). HMPV was identified in 30 cases in S1 and in 19 in the same period of S2 (36.6% reduction), whereas AdV was detected in 23 infants in S1 and in 7 in S2 (69.5% reduction). However, as the total number of infections was significantly lower in S2, the relative frequency of these viruses did not reach statistical significance. Conversely, the relative frequency of HRV and HBoV almost tripled in S2 (*p* < 0.001 and *p* = 0.013 respectively), although the absolute frequencies were similar among the two seasons. No significant differences were detected in the prevalence of other viruses or in the occurrence of viral coinfections.

### 3.2 Comparison of Clinical and Virological Characteristics of Respiratory Infections in Infants Who Received Nirsevimab (*N* = 63) Versus Infants Who Did Not (*N* = 126) in S2 Period

#### 3.2.1 Clinical Features

Table 2 presents a summary of the clinical characteristics of infants receiving nirsevimab compared to those not receiving nirsevimab during the S2 period. Infants in the nirsevimab group were younger at admission (*p* < 0.001), had a lower frequency of fever (*p* = 0.014), experienced fever for a shorter duration (*p* = 0.022), required high‐flow oxygen less frequently (*p* = 0.003), and had a lower rate of antibiotic prescriptions (*p* = 0.037). Although the difference was not statistically significant, ICU admissions tended to be less frequent in those treated with nirsevimab (*p* = 0.07).

**TABLE 2:** Comparison of clinical and virological characteristics of respiratory infections in nirsevimab‐immunized and nonimmunized infants during the postnirsevimab period (S2/postnirsevimab: October 1, 2023, to March 31, 2024).

	Nirsevimab	Nonnirsevimab	*p* value	Odds ratio (95% confidence interval)
	*N* = 63	*N* = 126		
Clinical features				
Sex (male)	43 (68%)	77 (63%)	0.446	1.3 (0.7–2.4)
Prematurity	9 (19%)	19 (18%)	0.902	1.1 (0.4–2.5)
Age (months)*	3.3 ± 2.7	5.9 ± 3.6	< 0.001	
Age < 1 month	8 (13%)	6 (5%)	0.05	2.8 (0.9–8.6)
Age < 6 months	54 (86%)	69 (56%)	< 0.001	4.7 (2.1–10.3)
Fever ≥ 38°C	23 (36%)	65 (53%)	0.035	0.5 (0.3–0.9)
Duration of fever (days)*	2.1 ± 1.1	2.9 ± 2.3	0.022	
Hypoxia, oxygen saturation < 95%	53 (84%)	103 (85%)	0.858	0.9 (0.4–2.1)
Duration of hypoxia (days)*	4.3 ± 3.1	4.6 ± 2.5	0.274	
Duration of stay > 5 days	17 (27%)	36 (29%)	0.744	0.9 (0.4–1.8)
Duration of stay (days)*	4.4 ± 3.1	4.9 ± 2.7	0.474	
High oxygen flow	26 (41%)	78 (65%)	0.003	0.4 (0.2–0.7)
Duration of high oxygen flow (days)*	3.8 ± 2.8	4.3 ± 2.0	0.263	
Intensive care unit admission	6 (9%)	24 (20%)	0.070	0.4 (0.2–1.1)
Duration of intensive care unit admission (days)*	3.5 ± 1.0	3.4 ± 1.6	0.953	
Mechanical ventilation	9 (16%)	17 (14%)	0.721	1.2 (0.5–2.8)
Antimicrobial treatment	13 (21%)	43 (35%)	0.037	0.5 (0.2–0.9)
Chest infiltrate	16 (25%)	35 (29%)	0.112	1.5 (0.6–3.8)
Diagnosis				
Bronchiolitis	43 (74.1%)	72 (64.3%)	0.434	
Recurrent wheezing	10 (17%)	27 (24%)		
Pneumonia	4 (7%)	12 (10.7%)		
Laryngitis	1 (1.7%)	1 (0.9%)		
Viral detection				
RSV	11 (18%)	62 (50%)	< 0.001	0.2 (0.1–0.4)
HRV	16 (26%)	39 (32%)	0.407	0.4 (0.4–1.5)
HBoV	2 (3%)	7 (6%)	0.462	0.5 (0.1–2.7)
HMPV	9 (14%)	10 (8%)	0.177	1.9 (0.7–5.0)
AdV	0	6 (6%)	0.050	Not available
Influenza	1 (2%)	6 (45%)	0.272	0.3 (0.1–2.7)
PIV	6 (10%)	2 (2%)	0.008	6.8 (1.3–35)
HCoV	6 (10%)	3 (2%)	0.031	4.3 (1.0–17.7)
Coinfection	4 (8%)	20 (18%)	0.098	0.4 (0.1–1.2)
Overall viral detection	40 (73%)	107 (88%)	0.014	0.4 (0.2–0.8)

* Mean and standard deviation.

Abbreviations: AdV, adenovirus; HBoV, human bocavirus; HCoV, human coronavirus; HMPV, human metapneumovirus; HRV, rhinovirus; PIV, parainfluenza virus; RSV, respiratory syncytial virus.

#### 3.2.2 Viral Detection

Overall, the frequency of viral detection was significantly lower in the nirsevimab group compared to the nonnirsevimab one (40 cases vs. 107, 63% reduction), as was the frequency of RSV identification (11 vs. 62 cases, 82% reduction) and AdV (0 vs. 6 cases). No differences were observed regarding the other respiratory viruses or the rate of viral coinfections.

Similar monthly distribution of respiratory admissions was observed in patients who received nirsevimab and those who did not (Figure 2b).

### 3.3 Comparison of Clinical Characteristics of RSV Infections (*N* = 409) in Nirsevimab (*N* = 11) Versus Nonnirsevimab Infants (*N* = 398)

#### 3.3.1 Clinical Features

Out of 63 infants who received nirsevimab, 11 (17.4%) were diagnosed with RSV‐LRTI. The median age at nirsevimab administration in RSV patients was 50 days (IQR: 1.5–136) and the mean interval between nirsevimab administration and admission was 46 days (IQR: 27–132). The age at admission was comparable between both groups, with similar proportion of admitted patients under 3 months old. Bronchiolitis was the most frequent diagnosis in both groups. Lower rates of fever (*p* = 0.001) and a reduced need for high‐flow oxygen (38.5% vs. 74.4%, *p* = 0.008) were observed in patients who received nirsevimab. The incidence and duration of hypoxia, as well as the length of hospitalization, were comparable between the groups. No significant differences were noted in terms of mechanical ventilation, infiltrate/atelectasis, or antibiotic use (Table 3).

**TABLE 3:** Comparison of clinical characteristics of respiratory syncytial virus infections between nirsevimab‐immunized and nonimmunized infants.

RSV infections *N* = 409				
	Nirsevimab	Nonnirsevimab	*p* value	Odds ratio (95% confidence interval)
	*N* = 11	*N* = 398		
Sex (male)	8 (73%)	245 (62%)	0.452	1.7 (0.4–6.4)
Prematurity	1 (9%)	52 (16%)	0.544	0.5 (0.1–4.2)
Age (months)**	4.0 (1.3–6.6)	3.0 (1.5–6.1)	0.932	
Age < 1 month	2 (18%)	63 (16%)	0.833	1.2 (0.2–5.6)
Age < 3 months	5 (45%)	199 (50%)	0.766	0.8 (0.2–2.8)
Fever ≥ 38°C	1 (9%)	172 (43%)	0.024	0.1 (0.1–1.0)
Hypoxia, oxygen saturation < 95%	10 (91%)	365 (92%)	0.925	0.9 (0.1–7.2)
Duration of hypoxia (days)**	4.0 (1.5–6.5)	5.0 (3.0–7.0)	0.190	
Duration of stay > 5 days	4 (31%)	156 (48%)	0.270	0.5 (0.1–1.6)
Duration of stay (days)**	5.0 (3.0–5.0)	5.0 (3.0–7.0)	0.323	
High oxygen flow	3 (27%)	283 (71%)	0.002	0.1 (0.1–0.6)
Duration of high oxygen flow (days)**	4.0 (3.0–4.0)	4.0 (3.0–6.0)	0.959	
Intensive care unit admission	3 (27%)	111 (28%)	0.964	0.9 (0.2–3.7)
Mechanical ventilation	3 (27%)	92 (23%)	0.762	1.2 (0.3–4.7)
Antimicrobial treatment	3 (27%)	113 (29%)	0.919	0.9 (0.2–3.6)
Chest infiltrate	2 (18%)	108 (27%)	0.857	0.8 (0.1–6.0)
Diagnosis				
Bronchiolitis	8 (80%)	327 (84%)	0.504	
Recurrent wheezing	2 (20%)	41 (10%)		
Pneumonia	0	21 (5%)		

** Median with interquartile range.

#### 3.3.2 Viral Detection

Among nonnirsevimab patients, the peak incidence of RSV admissions occurred primarily between October and December, with the highest rate observed in November (40%). In contrast, RSV infections in nirsevimab‐treated infants were more evenly distributed from October to March (*p* = 0.001). The frequency of RSV coinfections with other respiratory viruses was similar between the two groups (31% vs. 14%, *p* = 0.119).

## 4 Discussion

The primary objective of this prospective study was to evaluate the impact of nirsevimab immunization, not only on the frequency of hospitalizations related to RSV but also on those associated with other respiratory viruses in real‐world setting. Additionally, the study aimed to compare the clinical and epidemiological characteristics of hospital admissions for viral respiratory infections in infants under 12 months before and after the introduction of nirsevimab. Our findings demonstrated a significant reduction in hospitalizations due to viral respiratory infections, as well as a decrease in their duration, among infants under 1 year of age in the latter season when nirsevimab was implemented. Additionally, there was a decrease in ICU admissions and RSV‐related hospitalizations when comparing the 2023/2024 and 2022/2023 RSV epidemic seasons. These findings are in line with those observed in two clinical trials. The MELODY clinical trial, in healthy infants, reported an efficacy of 76.8% against RSV hospitalization and 78.6% against severe cases [9]. The HARMONY trial, in conditions that approximated real‐world settings, described a nirsevimab efficacy of 83.2% in preventing hospitalization for RSV‐associated LRTI and 75.7% in preventing very severe cases [10].

Previous population‐based studies conducted in Spain have shown a high acceptance rate of nirsevimab, with immunization rates around 90% [9, 10, 11, 12]. These studies reported similar rates of reduction in RSV‐related hospitalizations to ours. Ares‐Gómez et al. [13], in Galicia, found an effectiveness of 69% against all‐cause LRTI hospitalizations, which is quite similar to our reduction, and an effectiveness of 89.8% against RSV‐related LRTI hospitalizations and 86.9% against severe RSV‐related LRTI requiring oxygen support. Also in Spain, Lopez‐Lacort et al. [11], in a study performed in nine hospitals across three Spanish regions, reported effectiveness of 84% and 70% of nirsevimab against RSV‐related LRTI hospitalizations depending on the study design but found a lack of protection against non‐RSV hospitalizations. Mazagatos et al. [14] analyzed data from the Surveillance System of Acute Respiratory Infections in Spain (SiVIRA) and found a 75% relative reduction in the risk of RSV hospitalization. Lastly, Ezpeleta et al. [12] in another population‐based study in Navarra (Spain) estimated an effectiveness of nirsevimab in preventing RSV hospitalizations of 88.7%. Unlike other published studies [15, 16], in our hospital‐based study, we found a reduction not only in RSV hospitalizations after the introduction of nirsevimab but also a notable decrease in admissions due to other respiratory viruses such as HMPV (36.6%) and AdV (69.5%). Ares‐Gómez et al. [13] also reported substantial protection against all‐cause LRTI hospitalizations, with a global effectiveness estimate of 69.2%, although without detailing the reduction of each virus. To the best of our knowledge, this is the first report examining the impact of nirsevimab on the rate of non‐RSV LRTI admissions, revealing a significant decrease in HMPV‐ and AdV‐related hospitalizations following the introduction of this immune prophylaxis. This intriguing finding, coupled with the unchanged frequency of other viruses such as HRV and HBoV, points to a combination of direct and indirect factors influencing viral transmission dynamics and hospitalization patterns. Although nirsevimab is not directly active against other viruses, its introduction may have impacted HMPV and adenovirus infections through indirect mechanisms. By preventing RSV infections, indirectly affecting the dynamics of other viruses, nirsevimab may have lowered the risk of secondary infections with HMPV or AdV, which often cocirculate with RSV [17, 18]. Furthermore, RSV and HMPV often compete for the same ecological niche within the respiratory tract. With fewer RSV cases, the replication dynamics and transmissibility of HMPV may have been impaired [19]. Also, with RSV cases declining, some HMPV or AdV infections that previously warranted hospitalization might now present as milder cases managed in outpatient settings, altering hospital‐based surveillance data. In contrast, HRV and HBoV exhibit distinct epidemiological patterns. HRV is less seasonally dependent and highly stable in the environment [20], while HBoV frequently causes asymptomatic or mild infections [21], which may explain why their prevalence remained unchanged. Nevertheless, future research should aim to clarify these complex interactions through prospective studies, enhanced viral surveillance, and modeling of viral dynamics.

As previously mentioned, several epidemiological, population‐based studies have evaluated the impact of nirsevimab on the frequency of RSV infections in children, both in primary care and in hospitalized settings. However, few hospital‐based studies have evaluated the clinical characteristics of infants hospitalized with RSV or other respiratory viruses following the introduction of this preventive measure. Our results indicated that infants in S2 were older at admission, with a significantly smaller proportion of infants less than 1 month old than in S1. These results align with those of Ernst et al. [22], in Luxembourg, who also found a different age structure between 2023/2024 and 2022/2023 RSV seasons, with an increased average age of admitted children, probably attributable to the effect of administering nirsevimab shortly after birth.

Overall, the severity of LRTI significantly decreased after the introduction of nirsevimab. In our study, the length of hospital stays for LRTI admissions was notably shorter, decreasing from a mean of 5.8 days in S1 to 4.5 days in S2. As a result, the overall likelihood of requiring a hospital stay longer than 5 days was reduced by half in S2, with an even more substantial reduction among infants under 3 months of age, where the decrease was 70%. Our results also point to a reduction in ICU admissions for severe LRTIs after the introduction of nirsevimab, as has been described in other real‐world studies [11, 16, 23, 24]. These data highlight the huge impact of this preventive strategy in reducing the number of severe LTRI in infants, which, before nirsevimab, was one of the most significant challenges faced by healthcare systems worldwide.

Among children admitted for RSV‐LRTI, and despite only 11 RSV‐infected infants receiving nirsevimab prior to admission, a significant reduction in the need for high‐flow oxygen therapy was observed. These findings, consistent with those reported by Ernest et al. [22], suggest that nirsevimab not only significantly reduces hospitalizations for RSV‐LRTI but also may decrease their severity. However, given the current limited number of RSV cases, it will be crucial to reevaluate these results in future seasons to confirm and validate these observations.

The main limitation of our study is the small number of patients hospitalized after the introduction of nirsevimab, which is a result of the effectiveness of this preventive strategy. This decrease was particularly pronounced in the case of RSV infections, preventing stratified subgroup analyses. Additionally, our study is observational and descriptive rather than a clinical trial; therefore, our results cannot establish causality. This study also boasts several strengths. Our study period encompasses the entire 2022–2023 and 2023–2024 RSV epidemic waves. Clinical data were collected prospectively, and virological diagnosis was performed not only for RSV but also for 16 other respiratory viruses, allowing us to evaluate the impact of nirsevimab administration on all of them.

In conclusion, nirsevimab has proven to be highly effective in protecting young infants against severe outcomes associated with RSV infections. Furthermore, our study indicates that nirsevimab is also linked to a notable reduction in hospitalizations due to infections caused by other respiratory viruses, such as HMPV and AdV. To validate these findings and better understand its broader impact, it is essential to continue monitoring nirsevimab's effectiveness in future seasons. Confirming these results would further enhance its value as a preventive measure, underscoring its potential to provide significant prophylactic benefits.

## Author Contributions

**María Luz García‐García:** conceptualization, investigation, funding acquisition, writing – original draft, methodology, validation, visualization, writing – review and editing, software, formal analysis, project administration, data curation, supervision, resources. **Patricia Alonso‐López:** conceptualization, investigation, writing – review and editing, methodology, data curation, supervision, writing – original draft, visualization, formal analysis, validation, software. **Sonia Alcolea:** conceptualization, investigation, writing – review and editing, data curation, supervision, visualization, formal analysis, methodology. **M. Arroyas:** conceptualization, investigation, writing – review and editing, methodology, data curation, supervision, visualization, writing – original draft, formal analysis. **Francisco Pozo:** conceptualization, investigation, funding acquisition, data curation, resources, methodology, supervision, formal analysis, project administration, writing – review and editing, visualization, validation, software. **Inmaculada Casas:** resources, data curation, supervision, conceptualization, investigation, funding acquisition, writing – review and editing, methodology, formal analysis, project administration, visualization, validation, software. **Maria Iglesias‐Caballero:** investigation, visualization, writing – review and editing, data curation, supervision. **Rocío Sánchez‐León:** data curation, supervision, writing – review and editing, investigation, visualization. **Jara Hurtado‐Gallego:** investigation, visualization, writing – review and editing, data curation, supervision. **Cristina Calvo:** conceptualization, investigation, funding acquisition, writing – original draft, writing – review and editing, visualization, validation, methodology, software, formal analysis, project administration, data curation, supervision, resources.

## Conflicts of Interest

The authors declare no conflicts of interest.

### Peer Review

The peer review history for this article is available at https://www.webofscience.com/api/gateway/wos/peer‐review/10.1111/irv.70105.

## Data Availability

The data presented in this study are available upon request from the corresponding author.
